# Supplementary material for: Matching patients to clinical trials with large language models
Source: Nat Commun. 2024 Nov 18;15:9074. doi: 10.1038/s41467-024-53081-z (PMC11574183; doi:10.1038/s41467-024-53081-z)
Supplement: Supplementary file 1 — Supplementary Information [file 41467_2024_53081_MOESM1_ESM.pdf]

Supplementary Information for

Matching Patients to Clinical Trials with Large Language Models

Supplementary Table 1. Error analysis of the TrialGPT predictions. E1: Incorrect reasoning; E2: Lack of medical knowledge; E3: Ambiguous label definition; E4: other errors. (I) and (E) stand for inclusion and exclusion criteria, respectively.

	%	Example Criterion	TrialGPT Explanation	Comment
**E1**	30.7	(I) Patient at least 18 years of age presenting with a symptomatic chronic subdural hematoma	The patient is 85 years old, which meets the age requirement. However, there is no mention of the patient having a symptomatic chronic subdural hematoma. The patient had a car accident 3 weeks prior and had a normal head CT at that time, which suggests that he does not have a chronic subdural hematoma.	The manifestation is very likely symptomatic chronic subdural hematoma (elderly with history of accident, not shown initially but slowly developed symptoms potentially due to compression from blood accumulation).
**E2**	15.4	(E) Had been performed central venous puncture or	The patient note mentions that the patient underwent cardiac catheterization via	A femoral artery catheterization (via artery to coronary

		catheterization before.	the right femoral artery. This indicates that the patient has had a central venous puncture or catheterization before, which meets the exclusion criterion.	artery) is not CVC puncture / catheterization (via vein to central vein).
**E3**	26.9	(E) GeneXpert MTB/RIF sputum TB positive (China subjects only).	This criterion is not applicable as the patient's nationality or location is not mentioned in the note.	Should be “Not enough information”.
**E4**	26.9	(I) Subjects with visible skin disease, tattoos, skin condition, or abnormal skin color.	The patient note mentions that the patient has lesions on her neck, which can be considered a visible skin disease. Therefore, the patient does not meet this criterion.	Self-conflicting.

Supplementary Table 2. The prompt template for TrialGPT-Retrieval to generate a list of keywords for the given patient. {PATIENT_NOTE} denote the input patient note text.

You are a helpful assistant and your task is to help search relevant clinical trials for a given patient description. Please first summarize the main medical problems of the patient. Then generate up to 32 key conditions for searching relevant clinical trials for this patient. The key condition list should be ranked by priority. Please output only a JSON dict formatted as Dict{{"summary": Str(summary), "conditions": List[Str(condition)]}}.<br><br>Here is the patient description:<br><br>{PATIENT_NOTE}<br><br>JSON output:

Supplementary Table 3. The prompt template for TrialGPT to predict criterion-level eligibility for inclusion criteria. {PATIENT_NOTE} and {CLINICAL_TRIAL} denote the input patient note text and the clinical trial criteria, respectively.

You are a helpful assistant for clinical trial recruitment. Your task is to compare a given patient note and the inclusion criteria of a clinical trial to determine the patient's eligibility at the criterion level.<br>The factors that allow someone to participate in a clinical study are called inclusion criteria. They are based on characteristics such as age, gender, the type and stage of a disease, previous treatment history, and other medical conditions.<br>You should check the exclusion criteria one-by-one, and output the following four elements for each criterion:<br>Element 1. For each exclusion criterion, think step-by-step and generate your reasoning process: First, judge whether the criterion is not applicable (not very common), where the patient does not meet the premise of the criterion. Then, check if the patient note contains direct evidence. If so, judge whether the patient meets or does not meet the criterion. If there is no direct evidence, try to infer from existing evidence, and answer one question: If the criterion is true, is it possible that a good patient note will miss such information? If impossible, then you can assume that the criterion is not true. Otherwise, there is not enough information.

Element 2. If there is relevant information, you must generate a list of relevant sentence IDs in the patient note. If there is no relevant information, you must annotate an empty list.

Element 3. Classify the patient eligibility for this specific exclusion criterion: the label must be chosen from {"not applicable", "not enough information", "included", "not included"}. "not applicable" should only be used for criteria that are not applicable to the patient. "not enough information" should be used where the patient note does not contain sufficient information for making the classification. Try to use as less "not enough information" as possible because if the note does not mention a medically important fact, you can assume that the fact is not true for the patient. "included" denotes that the patient meets the inclusion criterion, while "not included" means the reverse.

You should output only a JSON dict exactly formatted as:

```dict{str(exclusion_criterion): list[str(element_1_step_by_step_reasoning), list[int(element_2_sentence_id)], str(element_3_eligibility_label)]}\n```

Here is the patient note, each sentence is led by a sentence_id:

{PATIENT_NOTE}

Here is the clinical trial:

{CLINICAL_TRIAL}
```

Plain JSON output:

Supplementary Table 4. The prompt template for TrialGPT to predict criterion-level eligibility for exclusion criteria. {PATIENT_NOTE} and {CLINICAL_TRIAL} denote the input patient note text and the clinical trial criteria, respectively.

You are a helpful assistant for clinical trial recruitment. Your task is to compare a given patient note and the exclusion criteria of a clinical trial to determine the patient's eligibility at the criterion level.<br>The factors that disqualify someone from participating are called exclusion criteria. They are based on characteristics such as age, gender, the type and stage of a disease, previous treatment history, and other medical conditions.<br>You should check the exclusion criteria one-by-one, and output the following four elements for each criterion:<br>Element 1. For each exclusion criterion, think step-by-step and generate your reasoning process: First, judge whether the criterion is not applicable (not very common), where the patient does not meet the premise of the criterion. Then, check if the patient note contains direct evidence. If so, judge whether the patient meets or does not meet the criterion. If there is no direct evidence, try to infer from existing evidence, and answer one question: If the criterion is true, is it possible that a good patient note will miss such information? If impossible, then you can assume that the criterion is not true. Otherwise, there is not enough information.

Element 2. If there is relevant information, you must generate a list of relevant sentence IDs in the patient note. If there is no relevant information, you must annotate an empty list.

Element 3. Classify the patient eligibility for this specific exclusion criterion: the label must be chosen from {"not applicable", "not enough information", "excluded", "not excluded"}. "not applicable" should only be used for criteria that are not applicable to the patient. "not enough information" should be used where the patient note does not contain sufficient information for making the classification. Try to use as less "not enough information" as possible because if the note does not mention a medically important fact, you can assume that the fact is not true for the patient. "excluded" denotes that the patient meets the exclusion criterion and should be excluded in the trial, while "not excluded" means the reverse.

You should output only a JSON dict exactly formatted as:

```dict{str(exclusion_criterion): list[str(element_1_step_by_step_reasoning), list[int(element_2_sentence_id)], str(element_3_eligibility_label)]}\n```

Here is the patient note, each sentence is led by a sentence_id:

{PATIENT_NOTE}

Here is the clinical trial:

{CLINICAL_TRIAL}
```

Plain JSON output:

Supplementary Table 5. TrialGPT aggregation prompt for generating the trial-level scores. {PATIENT_NOTE} and {CLINICAL_TRIAL} denote the input patient note text and the clinical trial criteria, respectively. {TrialGPT} denotes the criterion-level predictions made by TrialGPT (from Supplementary Tables 3 and 4).

You are a helpful assistant for clinical trial recruitment. You will be given a patient note, a clinical trial, and the patient eligibility predictions for each criterion.<br>Your task is to output two scores, a relevance score (R) and an eligibility score (E), between the patient and the clinical trial.<br>First explain the consideration for determining patient-trial relevance. Predict the relevance score R (0~100), which represents the overall relevance between the patient and the clinical trial. R=0 denotes the patient is totally irrelevant to the clinical trial, and R=100 denotes the patient is exactly relevant to the clinical trial.<br>Then explain the consideration for determining patient-trial eligibility. Predict the eligibility score E (-R~R), which represents the patient's eligibility to the clinical trial. Note that -R <= E <= R (the absolute value of eligibility cannot be higher than the relevance), where E=-R denotes that the patient is ineligible (not included by any inclusion criteria, or excluded by all exclusion criteria), E=R denotes that the patient is eligible (included by all inclusion criteria, and not excluded by any exclusion criteria), E=0 denotes the patient is neutral (i.e., no relevant information for all inclusion and exclusion criteria).

Please output a JSON dict formatted as Dict{"relevance_explanation": Str,

"relevance_score_R": Float, "eligibility_explanation": Str, "eligibility_score_E": Float}.

Here is the patient note:

{PATIENT_NOTE}

Here is the clinical trial description:

{CLINICAL_TRIAL}

Here is the criterion-level eligibility prediction:

{TrialGPT}

Plain JSON output:

Supplementary Note. Synthetic patient vignettes used in the case study.

Case 1

49 yo F; Esthesioneuroblastoma dx 2015; Kadish Stage C

Chemo RT Cisplat/Etop & 50 Gy /25 fx completed 8.5.15

Resection 10.15.15

Recurrence 2.7.21: Kadish Stage D; bone

3.5.21 L neck dissection

4.10.21 Cycle 1 Carbo, Etop

5.27.21 Cycle 2

Rescan in Jun: progression

Case 2

55yo M with metastatic stage IV anal cancer caused by HPV.

Current Diagnosis: Metastatic Anal SCC (squamous cell carcinoma) p16+

Date of Diagnosis: May 2021, stage T2N1aM0

Tumor molecular profile: Tempus xT: STK11, FAT1; TMB 7.4 mut/Mb, MSS

Tumor was removed in June 2021 followed by a month of radiotherapy and chemo (Mitomycin/Fluorouracil).

Metastasis identified in the liver in 03/2023

Received 2 lines of treatment to no success:

1st line 7/23-9/23 Chemo: Carboplatin/Paclitaxel

2nd line Immunotherapy: Pembrolizumab (Keytruda)

Case 3

We have a potential patient for your study. She is 55 y/o F w/ PMHx cervical cancer with metastatic disease to the peritoneum and bladder. She has received cisplatin/taxol/bevacizumab. She otherwise is in great condition (ECOG 0) and was interested in hearing from your group regarding your trial.

Case 4

History of Present Illness: 55 year-old man, non smoker, no EtOH abuse, with metastatic Nasopharynx cancer with abdominal LN in, EBV(+), AJCC 8th ed clinical stage III (cT2, cN1, cM0).

10/2018 Chemo and RT with Cis 40 weekly with RT -> adjuvant chemotherapy 08/2019 biopsy proven recurrence in portal LN with several other involved LN's on PET scan 10/2019 Palliative Carbo/Gemcitabine 03/2020 palliative Pembrolizumab for progression of disease 06/2020 Progression of disease on PET in liver and LN's -> 07/2020 palliative docetaxel -> 01/2021 progression of disease in liver and lungs

Patient reports occasional decreased hearing, denies tinnitus, persistent neuropathy

Oncology Hx:

5/2018 Initial Diagnosis

7/2018

CT Neck bilateral supraclavicular, posterior cervical triangle, and submandibular adenopathy Nodes measure up to 15 mm short axis in the right supraclavicular region, 16 mm short axis in the right posterior cervical triangle, and 14 mm short axis in the right submandibular region. Nodes measure up to 11 mm short axis in the left supraclavicular region, 14 mm short axis in the lelt posterior cervical triangle, and 14 mm short axis in the left submandibular region

8/2018

Blopsy

FNA: Cervical lymph node metastatic carcinoma.

9/2018

Interventional/Imaging

PET CT scan intense nasopharyngeal uptake measuring 35mm (max SUV 10.6). Multiple bilateral hypermetabolic retropharyngeal and cervical nodes. Compatible with nodal metastases (N3).

Biopsy

Bx Nasopharynx Nasopharyngeal carcinoma nonkeratinizing differentiated type.

Pancytokeratin - positive.

P40-positive

EBV ISH-positive P16-negative

Allergies:

Co-trimoxazole and penicillins class

Family History: reviewed, Social History: Reviewed

Current Medications:

Sodium Fluoride (SF) 1.1% Dent Gel

Gabapentin (NEURONTIN) 300 mg Oral Cap Take 1 capsule by mouth at bedtime as needed for pain

Lidocaine 5% Top Oint Apply to affected area(s) 2 times a day

Sodium Fluoride (SF 5000 Plus) 1.1% Dent Crea Brush at night time before bed. Do not rinse, eat, or drink for 30 minutes after.

Levothyroxine (LEVOTHROID/SYNTHROID) 50 mcg Oral Tab Take 1 tablet by mouth daily 30 minutes before breakfast

ECOG Performance status 0

ROS negative

Physical exam

General appearance alert, in no distress

Eyes pupils equal and reactive

Throat-normal

Chest-clear to auscultation, no wheezes, rales or rhonchi

Heart-regular rate and rhythm

Abdomen-soft, nontender, nondistended, no masses or organomegaly

Extremities no clubbing, cyanosis or edema

Laboratory Results:

WBC COUNT 10.9

Hgb 12.5 (L)

Hematocrit 39.4

Platelets count 388

ALT 39

AST 43 (H)

ALKALINE PHOSPHATASE 109

Bilirubin, total 0.6

Creatinine 0.8

Case 5

This is a 50-year-old female referred for the multidisciplinary management of newly diagnosed anal squamous cell carcinoma. 3 months ago, around August 2023, she started noticing stool coming through her vagina. She has had a history of sexual abuse and trauma and visits with doctors have been very anxiety provoking. She states that she has had constipation for around 10 years as well as intermittent hemorrhoids, which she treated herself and would use laxatives. She also admits to intermittent blood in her stool. She was diagnosed with a rectovaginal fistula and was found to have scar tissue in the rectum and was referred to Dr. Gearhart at Johns Hopkins for further management. At that time exam revealed suspicion for anal cancer and a colonoscopy and biopsy was scheduled. On 10/5/2023 a colonoscopy was performed and this revealed a half circumferential ulcerated mass found in the recturn the mass began in the anal canal and penetrated through the vagina. The mass was 4 x 3 cm in size and pathology revealed invasive squamous cell

carcinoma with focal keratinization. A CT scan of the chest abdomen pelvis was performed on 10/4/2023 at Johns Hopkins and this revealed a large lower rectal/anorectal with probable metastatic mesorectal and presacral lymphadenopathy, also suggestion of rectovaginal fistula, there was mediastinal adenopathy including a right paratracheal lymph node 15 mm and a subcarinal lymph node 13 mm and a left paraesophageal lymph node 7 mm. Patient underwent a diverting colostomy on 10/05/2023. A PET scan was obtained on 10/4/2023 and this revealed significant activity in the anal region SUV 17, significant activity in multiple mediastinal, perirectal, pelvic sidewall and inguinal lymph nodes SUV 10. The largest lymph node was the left inguinal region measuring 1.5 cm.

Currently she is status post diverting colostomy on 10/5/2023, she is recovering well, the colostomy is working well and is clean dry and intact. She has a high level of anxiety due to her history of sexual abuse and trauma, but she is functioning well and is processing this new diagnosis. She has no chest pain or shortness of breath, no abdominal pain, no lower extremity edema, no focal neurologic deficits. She has no constitutional symptoms such as weight loss or night sweats.

Most recent blood work on 10/5/2023: WBC 9.3 hemoglobin 10.6 hematocrit 32.5 platelets 200,000, creatinine 0.67

Interim history:

Mindy does not state any clinical worsening, overall she feels better than she did months ago She does not have any worsening shortness of breath or fatigue. Her ECOG status overall remains 1. She denies any bleeding from her ostomy bag.

Review of systems: 10/14 systems were reviewed and are negative except for the positive pertinents mentioned in the HPI.

Past Medical History:

Diagnosis

Cancer (CMS/HCC)

Family history of adverse reaction to anesthesia

FATHER HAS HAD 2 EPISODES OF INTEROPERATIVE AWARENESS

Past Surgical History:

Procedure

BRONCHOSCOPY 2022

COLON SURGERY diverting colostomy 10/5/2023

COLONOSCOPY 09/15/2023

COLOSTOMY 2023

DILATION AND CURETTAGE OF UTERUS 1994

Socioeconomic History

Tobacco Use

• Smoking status:

Former Smoker

Packs/day:

0.50

Years:

6.00

Pack years:

3.00

Current outpatient medications

Duloxetine

Gabapentin

Ibuprofen

Levothyroxine

Lorazepam

Methadone

Prochlorperazine

Tramadol

Potassium chloride

Allergies

Allergen - reactions

Tape – itchy

Lidocaine – rash

BP (!) 95/50 | Pulse 89 | Temp 36.8°C (Oral) | Resp 18 | Ht 162.6cm (64") | Wt 56.7 kg | SpO2 98% | BMI 21.46 kg/m^2

ECOG Performance Status: 1

General Appearance: appropriate for stated age

HEENT: anicteric sclera. Oropharynx clear without lesions.

Lymphatics: no palpable adenopathy.

Respiratory: clear to auscultation bilaterally without adventitious sounds.

Cardiovascular: Regular rate and rhythm with no added sounds. Skin: no lesions, rashes, ulcers, ecchymosis or bruises.

Musculoskeletal: no focal bone tenderness.

Neurological: no focal deficits. Mood, affect, and behavior are normal and appropriate.

Extremities: no clubbing, cyanosis or edema

Assessment and Plan

This a 50-year-old female diagnosed on 09/15/2023 via colonoscopy with invasive squamous cell carcinoma of the anus (4 x 3 cm mass, mesorectal/presacral

lymphadenopathy) complicated by rectovaginal fistula status post diverting colostomy 10/5/2023. Staged as cT3N1M0 stage IIIC.

Case 6

59yo man with history of HPV and anal condyloma, Stage | Anal Cancer s/p chemoradiation Jan 2015 presenting for 6 months RLQ pain found to have lymphadenopathy concerning for recurrence of anal cancer.

In October 2014 patient reported constipation and change in bowel habits associated with BRBPR. He underwent colonoscopy which revealed a mass 1.3cm from the rectum with biopsy positive for squamous cell carcinoma of the anus,. Patient underwent chemoradiation ending in January 2015. He has been monitored with annual anoscopy and imaging most recently 06/2018 notable for hepatomegaly and small subcentimeter cysts within the left kidney.

6/2018 PET/CT: Metabolically active confluent adenopathy at the aortic bifurcation extending RL common iliac nodes and R internal/external iliac nodes most c/w metastatic disease. No metabolic evidence of local recurrent disease in the anus. No other e/o dlstant metastatic disease.

Path: (A) Rectal promontory implant, resection for path:-Metastatic poorly differentiated squamous cell carcinoma with focal keratinization. (8) Retroaortic lymph node, resection, for frozen section and permanent:-Metastatic poorly differentiated squamous cell carcinoma with focal keratinization.

07/21/18-cisplatin/5-FU completed 4th cycle on 8/2/18 w/ a 35% PR in right common iliac lymphadenopathy

10/2018: RT to sacral lesion. Started on maintenance 5-FU and then Capecitabine which was discontinued on 6/19/2019.

CT scan 11/2019 with pelvic lymphadenopathy; IR biopsy positive for recurrence.

REVIEW OF SYSTEMS:

Constitutional

Normal-No fevers, chills, night sweats, excessive fatigue or weight loss.

Allergic/Immunologic

Normal-No reactions.

Head

Normal-No trauma or headaches.

ENMT

Normal-No problems with hearing, no sore throat, no sinus drainage.

Eyes

Normal-No significant visual difficulties. No diplopia.

Neck

Normal-No pain or swallowing problems.

lntegumentary

Normal-No chronic rashes, Inflammation, ulcerations or skin changes.

Cardiovascular

Normal-No anginal chest pain, palpitations or orthopnea.

Respiratory

Normal-No dyspnea on exertion, chest pain, cough or hemoptysis.

Gastrointestinal

Normal-No nausea, vomiting, diarrhea, GI bleeding, or constipation, No change in bowel habits, no heartburn or early satiety.

Genitourinary

Normal- No abnormal genital masses. No hematuria, hesitancy, incontinence, discharge or other problems with urination. Normal sexual function.

Musculoskeletal

Normal- L lower back pain

Neurologic

Normal-RLE numbness (Improving)

Psychiatric

Normal-No insomnia, depression, mania or mood swings.

Endocrine

Normal-No diabetes, thyroid disease or hormone replacement. No hot flashes or night sweats

Hematologic/Lymphatic

Normal. No easy bruising or bleeding. The patient denies any tender or palpable lymph nodes

PHYSICAL EXAMINATION:

Performance status: 0-Fully active, able to carry on all predisease activities without restrictions (ECOG)

Vital Signs:

Height-156.50 cm

Weight-44.3 kg (LOW)

BSA-1.40 sq.m

LABORATORY:

Glucose, Random (11761405)-83

BUN-20

Creatinine (2921236) -0.78

Creatinine Cl (Est) -55.3500

Sodium (2921248)-145

Potassium (2921259)-4.2

Chloride-105

CO2 (2921282)-28

Anion Gap-12

Calcium-9.6

Protein, Total (2921726) -7.3

Albumin-3.8

Globulin-3.5

A/G Ratio-1.1

SGOT/AST-11

SGPT/ALT-21

Alkaline Phosphatase - 75

Bilirubin, Total-0.4

WBC-4.5

RBC-3.85

HGB (4413459) - 12.0

HCT (2921549)-35.5

MCV-92.2

MCH-31.2

MCHC-33.8

RDW-12.6

Platelet (2921699)-291

MPV-9.1

Neutrophil %-69.6

Lymphocyte %-27.6

Monocyte %-7.1

Eosinophil %-4.2

Basophil % -1.3

Immature Grans %-0.2

Neutrophils-2.7

Lymphocytes 1.2

Monocytes -0.3

Eosinophils-0.2

Basophils-0.1

Immature, Grans #-0.01

NRBC-0

NRBC (Abs) -0.0
