# Supplementary material for: Matching patients to clinical trials with large language models
Source: Nat Commun. 2024 Nov 18;15:9074. doi: 10.1038/s41467-024-53081-z (PMC11574183; doi:10.1038/s41467-024-53081-z)
Supplement: Supplementary file 2 — Reporting Summary [file 41467_2024_53081_MOESM2_ESM.pdf]

nature portfolio

Corresponding author(s): Zhiyong Lu, PhD

Last updated by author(s): Aug 21, 2024

Reporting Summary

Nature Portfolio wishes to improve the reproducibility of the work that we publish. This form provides structure for consistency and transparency in reporting. For further information on Nature Portfolio policies, see our Editorial Policies and the Editorial Policy Checklist.

Statistics

For all statistical analyses, confirm that the following items are present in the figure legend, table legend, main text, or Methods section.

n/a	Confirmed	
☐	☒	The exact sample size (*n*) for each experimental group/condition, given as a discrete number and unit of measurement
☐	☒	A statement on whether measurements were taken from distinct samples or whether the same sample was measured repeatedly
☐	☒	The statistical test(s) used AND whether they are one- or two-sided<br>*Only common tests should be described solely by name; describe more complex techniques in the Methods section.*
☒	☐	A description of all covariates tested
☒	☐	A description of any assumptions or corrections, such as tests of normality and adjustment for multiple comparisons
☐	☒	A full description of the statistical parameters including central tendency (e.g. means) or other basic estimates (e.g. regression coefficient) AND variation (e.g. standard deviation) or associated estimates of uncertainty (e.g. confidence intervals)
☐	☒	For null hypothesis testing, the test statistic (e.g. *F*, *t*, *r*) with confidence intervals, effect sizes, degrees of freedom and *P* value noted<br>*Give P values as exact values whenever suitable.*
☒	☐	For Bayesian analysis, information on the choice of priors and Markov chain Monte Carlo settings
☒	☐	For hierarchical and complex designs, identification of the appropriate level for tests and full reporting of outcomes
☒	☐	Estimates of effect sizes (e.g. Cohen's *d*, Pearson's *r*), indicating how they were calculated

Our web collection on statistics for biologists contains articles on many of the points above.

Software and code

Policy information about availability of computer code

Data collection	To evaluate TrialGPT, we use three publicly available cohorts of 183 patients with over 75,000 trial eligibility annotations: the SIGIR 2016 cohort, the TREC 2021 Clinical Trial cohort, and the TREC 2022 Clinical Trial cohort. They are publicly available at https://data.csiro.au/collection/csiro:17152, https://www.trec-cds.org/2021.html, and https://www.trec-cds.org/2022.html, respectively. As a pilot case study, we also collected 6 semi-synthetic patient summaries for 6 clinical trials, which are available in the supplementary materials.
Data analysis	We conducted all the data analysis in Python (version 3.9.13) using Scipy (version 1.11.4) for the data analysis. We used the openai package (version 1.30.5) in Python to call the AzureOpenAI API. Our data analysis code is publicly available at: https://github.com/ncbi-nlp/TrialGPT.

For manuscripts utilizing custom algorithms or software that are central to the research but not yet described in published literature, software must be made available to editors and reviewers. We strongly encourage code deposition in a community repository (e.g. GitHub). See the Nature Portfolio guidelines for submitting code & software for further information.

Data

Policy information about availability of data

All manuscripts must include a data availability statement. This statement should provide the following information, where applicable:

- Accession codes, unique identifiers, or web links for publicly available datasets
- A description of any restrictions on data availability
- For clinical datasets or third party data, please ensure that the statement adheres to our policy

The TREC Clinical Trial 2021 and 2022 cohorts can be downloaded from http://www.trec-cds.org/2021.html and http://www.trec-cds.org/2022.html, respectively.

The SIGIR cohort is publicly available at https://data.csiro.au/collection/csiro:17152. The clinical vignettes for the user study are available in the Supplementary Materials. The criterion-level annotations generated in this study have been deposited in the Hugging Face database under accession code: https://huggingface.co/datasets/ncbi/TrialGPT-Criterion-Annotations. Preprocessed data files generated in this study have been deposited in the GitHub database under accession code: https://github.com/ncbi-nlp/TrialGPT. Source data are provided with this paper.

Research involving human participants, their data, or biological material

Policy information about studies with human participants or human data. See also policy information about sex, gender (identity/presentation), and sexual orientation and race, ethnicity and racism.

Reporting on sex and gender	We used publicly available synthetic patient summaries and reported their biological sex distribution in Table 1.
Reporting on race, ethnicity, or other socially relevant groupings	n/a. We used publicly available synthetic patient summaries.
Population characteristics	n/a. We used publicly available synthetic patient summaries.
Recruitment	n/a. We used publicly available synthetic patient summaries.
Ethics oversight	n/a. We used publicly available synthetic patient summaries.

Note that full information on the approval of the study protocol must also be provided in the manuscript.

Field-specific reporting

Please select the one below that is the best fit for your research. If you are not sure, read the appropriate sections before making your selection.

☒ Life sciences ☐ Behavioural & social sciences ☐ Ecological, evolutionary & environmental sciences

For a reference copy of the document with all sections, see nature.com/documents/nr-reporting-summary-flat.pdf

Life sciences study design

All studies must disclose on these points even when the disclosure is negative.

Sample size	183. We did not perform a sample size calculation. We used all publicly available patient cases that have been annotated for clinical trial eligibility. While there are only 183 unique patients, each has over 400 trial annotations on average. In total, there are over 75,000 patient-trial eligibility annotations, with which we got statistically significant results.
Data exclusions	We excluded two notes in the SIGIR cohort: one note describes a group of people (not a single patient), and another one is not associated with any eligibility annotations. This is ad-hoc exclusion and the criteria were not pre-established. We have documented it in the Methods section.
Replication	n/a. Replications are not applicable because the computational results are deterministic without randomness.
Randomization	n/a; not an interventional study
Blinding	n/a; not an interventional study

Reporting for specific materials, systems and methods

We require information from authors about some types of materials, experimental systems and methods used in many studies. Here, indicate whether each material, system or method listed is relevant to your study. If you are not sure if a list item applies to your research, read the appropriate section before selecting a response.

Materials & experimental systems

n/a	Involved in the study	
☒	☐	Antibodies
☒	☐	Eukaryotic cell lines
☒	☐	Palaeontology and archaeology
☒	☐	Animals and other organisms
☒	☐	Clinical data
☒	☐	Dual use research of concern
☒	☐	Plants

Methods

n/a	Involved in the study	
☒	☐	ChIP-seq
☒	☐	Flow cytometry
☒	☐	MRI-based neuroimaging

Plants

Seed stocks	n/a
Novel plant genotypes	n/a
Authentication	n/a
